# The Effects of Acupuncture on Bladder Interstitial Cells of Cajal Excitability in Rats with Overactive Bladder

**DOI:** 10.1155/2013/261217

**Published:** 2013-09-30

**Authors:** Qi-fan Feng, Yuen-hao Hou, Wen-guang Hou, Zhi-xian Lin, Kang-min Tang, Yue-lai Chen

**Affiliations:** Shanghai University of Traditional Chinese Medicine, Shanghai 201203, China

## Abstract

It is well known that acupuncture treatment has an effect on patients with an overactive bladder, but the mechanism of its action remains to be clarified. This study was aimed to investigate the effects of acupuncture on bladder overactivity, and the excitability of interstitial cells of Cajal of the bladder in a rat model of partial bladder outlet obstruction. Electroacupuncture (continuous wave, 30 Hz, 1 mA) was applied to stimulate the Ciliao point (BL32) and the Huiyang point (BL35) of rats for 20 min, 3 days. Results showed that acupuncture suppressed detrusor unstable contraction frequency and decreased detrusor maximum pressure in the bladder filling period. Compared with the normal control rats, HCN2 mRNA and protein expression within the bladder were upregulated and were reversed by electroacupuncture in overactive bladder rats as determined by RT-PCR, western blotting and immunohistochemistry. Moreover, in-vitro cell-cultured OAB rats bladder interstitial cells of Cajal intracellular Ca^2+^ concentration were higher than normal control rats, which were lowered after acupuncture treatment. These findings suggest that acupuncture stimulation can suppress bladder overactivity, and regulate the excitability of bladder interstitial cells of Cajal in treatment of overactive bladder myogenic mechanism.

## 1. Introduction

Overactive bladder (OAB) syndrome is characterized by urinary frequency and urgency with or without urge incontinence, and is often accompanied by nocturia. Acupuncture, as one of the traditional Chinese therapeutics, provides a nondrug therapy for OAB. It has been reported that the acupuncture stimulation to the sacral vertebrae has a suppressive effect on bladder activity, improves the symptom of nocturnal enuresis, decreases mean urge frequency, and diminishes voiding symptoms [1–5]. Our previous clinical studies have showed that acupuncture could improve urine dynamics condition, inhibit detrusor instability contraction, increase the bladder capacity, and improve quality of life significantly in OAB patients [6–8]. Although the effect of acupuncture on bladder activity has been confirmed, the mechanism of its action remains to be clarified. We previously found that the nitrergic neurotransmitter in bladder neck and detrusor was obviously decreased in rats with unstable bladder, the electroacupuncture treatment could significantly increase the contains of NOS in bladder tissue, regulate the bladder function, and inhibit bladder overactivity [9].

Many studies have found that interstitial cells of Cajal (ICCs) in bladder, like in gastrointestinal tract, act as primary pace maker cells that generate depolarizing currents into neighboring smooth muscles and coordinate muscle contractions, playing a fundamental role in signal transmission from bladder nerves to smooth muscle cells [10]. Bladder generates intrinsic autonomic contractions, and these intrinsic contractions are coordinated by the specialized system of ICCs [11]. c-Kit is used as an identification marker of ICCs, and Glivec (imatinib mesylate) is a c-kit receptor inhibitor, which reduces bladder overactivity. ICCs are linked to each other and to smooth muscle cells by gap junctions forming a continuous network, producing a syncytium of neurons enabling fast signal transduction [12]. c-Kit-positive ICCs are more numerous in human OAB detrusor than normal detrusor, suggesting that bladder ICCs one associated with the pathophysiology of OAB [13]. Hyperpolarization-activated cyclic nucleotide-gated (HCN) channels are unique among vertebrate voltagegated ion channel, which lead to activation on hyperpolarization. The HCN cation channel protein is regarded as the structural basis of the hyperpolarization-activated inward (Ih) current, which plays a very important role in pacemaking [14]. HCN protein is identified in ICCs of rat bladder, the expression of HCN protein is enhanced obviously in OAB rats, which may be responsible for the increased bladder excitability [15]. The change of structure and cell communication function of pace maker ICCs in bladder result in bladder overactivity. Whether the effect of acupuncture stimulation merely acts on the detrusor smooth muscle cells or to achieve through regulating the pace marker ICCs excitability? Therefore, the present study was performed in rats to examine: (1) the effects of acupuncture stimulation on bladder overactivity; (2) whether the change of bladder ICCs excitability mediates detrusor unstable contraction; (3) how acupuncture regulates the bladder ICCs excitability in treatment of unstable bladder myogenic mechanism.

## 2. Materials and Methods

### 2.1. Animals and Study Design

All experiments were performed on female Wistar rats, obtained from the Experimental Animal Center, Shanghai University of Tranditional Chinese Medicine, weighing 180–200 g at the beginning of the experiment. Animals were housed in a 12 h light/dark cycle with food and water available ad libitum. The room temperature was maintained at 22 ± 1°C and relative humidity at 45–50%. Five animals were housed in each cage. All experiments were performed complying with international ethical standards in the care and use of animals, and passed the examination of animal ethics committee of Shanghai University of TCM.

Female Wistar rats (*n* = 100) firstly were randomly divided into two groups: normal control (*n* = 15), model (*n* = 85). Rats were anesthetized by intraperitoneal injection of 1% pentobarbital sodium (50 mg/kg). The normal control group underwent a sham operation. Partial bladder outlet obstruction (PBOO), the classical method to induce OAB [16], was performed in model group. After 6 weeks, the models were assessed by cystometrogram under urethane anesthesia. When the detrusor contraction caused by pressure fluctuation shows phase contraction wave, the model was set up [17]. The success models (*n* = 46) were randomly divided into 3 groups: model group (*n* = 15), acupuncture group (*n* = 15), Glivec group (*n* = 16). Model group were no treatment, acupuncture group were treated by electroacupuncture. Glivec group were irrigated in the bladder with ICCs blocker Glivec (10^−5 ^mol/L, 1 mL/day, Santa Cruz Biotechnology), other groups were treated by bladder irrigation physiological saline, and every group was continuous treatment for 3 days.

### 2.2. Acupuncture Treatment

Conscious rats were treated by acupuncture. One acupuncture needle (diameter, 0.22 mm) was positioned almost vertically underneath the periosteum about 5 mm lateral to the midline of the S_2_ (BL32, Ciliao) and the other acupuncture needle (diameter, 0.22 mm) was positioned almost vertically underneath the periosteum about 5 mm lateral to the midline of the coccyx (BL35, Huiyang), bilateral symmetry. Stimulated electrically at both right and left points with frequency of 30 Hz and intensity of 1 mA, the entire procedure lasting 20 min. 

### 2.3. PBOO Operation and Cystometric Analysis

The operations of PBOO were performed under general anesthesia provided by intraperitoneal injection of 1% pentobarbital sodium (50 mg/kg). A 2-3 cm midline suprapubic skin incision was made, the fascia was reflected, the peritoneum was opened, and the bladder was identified. The urethra was inserted by a epidural catheter (diameter 1 mm), the proximal urethra was separated by a smooth forceps and tied with 2-0 sterile silk, the degree of tightness was that the catheter could be pulled easily, then the catheter was removed. The midline incision was closed in two layers with 5-0 polypropylene suture.

The models were assessed by cystometric analysis after 6 weeks. All Rats were anesthetized by intraperitoneal injection of 20% urethane (0.5 mL/100 g). One end of the transvesical catheter (polyethylene catheter-50) was inserted into the bladder through urethra and the other was connected to a pressure transducer and syringe pump via a 3-way stopcock to record intravesical pressure and infuse saline into the bladder. After the bladder was emptied, cystometry was performed with saline infused at 0.2 mL/min. The detrusor unstable contraction frequency and maximum pressure in the bladder filling period were recorded before and after treatment, the detrusor unstable contraction frequency was recorded by counting no-voiding contraction. 

### 2.4. RNA Extraction and Quantitative RT-PCR

All rats were killed by cervical dislocation, the abdomen was opened, trigone of urinary bladder was aseptically isolated and immediately frozen in liquid nitrogen, and placed immediately to be stored at −80°C until it is used. Each Sample was collected in 1 mL ice-cold Trizol (Invitrogen, Carlsbad, CA, USA). After its homogenization and sonification, RNA was extracted with chloroform, precipitated with isopropyl alcohol, and dissolved in 30 *μ*L RNAse-free DEPC. The RNA concentration and purity were analyzed by a Nanodrop spectrophotometer (Nanodrop Technologies, Wilmington, DE), with the spectral absorption at 260 and 280 nm. For cDNA synthesis, oligo(dT) primers, 1 *μ*g of each total RNA sample, and the RevertAid First Strand cDNA Synthesis Kit (Fermentas, Ontario, Canada) were used, following the guidelines of the manufacturer. cDNA samples were placed on ice and stored at −20°C until further use. Prior to the analysis, 10 *μ*L of each cDNA sample was diluted with 90 *μ*L of MilliQ water.

Quantitative RT-PCR was carried out in 20 *μ*L buffer solution containing 1 *μ*L of diluted cDNA sample, 10 *μ*L 2×SYBR green II qRT-PCR kit (Toyobo, Osaka, Japan), 1 *μ*L of each primer (5 *μ*M), and 8 *μ*L of MilliQ water. Primers to detect the mRNAs of the housekeeping gene *β*-actin of Hcn were designed using Primer 3 (http://frodo.wi.mit.edu/). Primer pairs were for *β*-actin: 5′-TCTGTGTGGATTGGTGGCTCT-3′ and 5′-AGAAGCATTTGCGGTGCAC-3′, for Hcn: 5′-ACCCCCAGCTCGTCTACTCT-3′ and 5′-ACCCCATCTTGTTTCTGCAC-3′. Cycling conditions were 10 min 95°C, followed by 40 cycles of 15 sec at 95°C and 1 min at 60°C. After cycling, a melting protocol was performed with 15 sec at 95°C, 1 min at 60°C, and 15 sec at 95°C, to control product specificity. The fold change (FC) in target gene cDNA relative to selected endogenous control gene was determined as follows: FC = 2^−ΔΔCt^, where ΔΔCt = (Ct_Target_ − Ct_Control_) test − (Ct_Target_ − Ct_Control_) control. Ct values were defined as the number of the PCR cycles at which the fluorescence signals were detected.

### 2.5. Western Blotting

Frozen tissues were homogenized in cold lysis buffer (Beyotime Biotechnology Co., Haimen, Jiangsu, China) and centrifuged at 13, 000 rpm for 5 min at 4°C, the supernatant total protein was quantified by Lowry method [18]. Samples (30 *μ*g total protein per loading) were separated on a 10% sodium dodecyl sulfate-polyacrylamide gel, electrophoresis gel, and electrotransfered onto polyvinylidene difluoride membranes (Millipore, Bedford, MA, USA). Themembranes were blocked with 5% nonfat milk overnight at 4°C, and thenincubated with the primary antibodies recognizing *β*-actin (mouse monoclonal, 1 : 5000, Abcam, Cambridge, UK) or HCN2 antibody (rabbit polyclonal,1 : 1000, Cell Signaling Technology, Danvers, MA, USA) for 2 h at 22°C. Then, the membranes were incubated with a horseradish peroxidase-conjugated antimouse (1 : 5000, Abcam, Cambridge, UK) or goat antirabbit HRP (1 : 4000, Abcam, Cambridge, UK) secondary antibodies. The signal was visualized with ECL plus reagent (GE healthcare, Buckinghamshire,UK) and exposed onto X-ray film (Kodak, Rochester, NY, USA). Protein ratios were calculated based on densitometrical quantification of scanned films in Image J software. HCN protein levels were normalized to actin levels and to levels of a control animal sample. 

### 2.6. Immunohistochemistry

The bladder was fixed in buffered 10% formalin for 24 h, dehydrated and embedded in paraffin, then cut section at 3 *μ*m for IF assay. In summary, slides were immersed to a boil (99°C~100°C) in 0.01 M sodium citrate buffer (pH 6.0) for 10 minutes. After the nonspecific binding was blocked with normal 5% goat serum, slides were incubated overnight with the primary antibodies c-Kit (H-300) antibody (sc5535, Santa Cruz Biotechnology) and Anti-HCN2 antibody (ab84817, Abcam), followed by the mixture of two fluorescent conjugated secondary antibodies for 30 min, Alexa Fluor 488 goat antirabbit IgG (Cat:A11034, Invitrogen, USA) and Alexa Fluor 555 goat anti-mouse IgG (Cat:A21424, Invitrogen, USA). Finally, the slides were mounted with DAPI mounting solution (P36935, Invitrogen, USA) and evaluated by 50i Nikon microscope in dark. The nucleus were visualized blue using a filter 330–380 nm and positive labeled expression with green by the filter 465–495 nm, with red by the filter 530–600 nm.

### 2.7. Primary Bladder ICCs Cell Culture and Identification

Referring to the previous experiments [19, 20], the rat bladder was dissected and placed in D-hanks' solution and the mucosal layer was removed. The smooth muscle was cut into small pieces and placed into 1 mg/mL collagenase type II (Worthington), 0.5 mg/mL trypsin (PAA), 1 mg/mL BSA (Huamei biological engineering company, China) solution. The smooth muscle was incubated in shaking bath for 8 min at 37°C 300 RPM, repeated 5 times. After incubation cells were centrifuged at 1500 RPM for 5 min, then the superfusate containing collagenase was removed. Cells were resuspended in 5 mL RPMI solution containing 30% FBS and centrifuged again at 1500 RPM for 5 min (twice). After the last cleaning process, 3 mL RPMI solution containing 30% FBS was added for the 1 plates (diameter 3 cm) having 2.5 mL cell suspension each, for measurement of intracellular-free Ca^2+^ concentration use 96 well plates. In our experiment, we found that while the ICCs took 3-4 hours to become attached to the plate of the culture disk, the detrusor cells would take 6 hours to do the same. Therefore, we used this time difference to separate these two types of cells concerned. Experiments were carried out on 1-2 days-old cultures. The ICCs in the cultures were morphologically distinct from the smooth muscle cells and their identity was confirmed with immunocytochemistry using primary antibodies against c-Kit. 

Immunohistochemistry to visualize cells expressing c-Kit immunoreactivity, cells were fixed in 3.7% formaldehyde (Thremo fish) 10 min, blocked in 1% bovine serum albumin (BSA) for 20 min, and preparations were loaded in the dark incubated for 12 h in PBS containing c-Kit protein (SC 5535, diluted 1 : 200, Santa Cruz Biotechnology). The cell plate was washed and then incubated for another 1 h 37°C in antirabbit IgG antibody labelled with a fluorescent marker (IgG-Alexa Fluor 488, diluted 1 : 900, Cell Signaling Technology). After washing with PBS, the preparations were observed using an inverted fluorescence microscopy (Axiovert 40 CFL, Zeiss, Germany).

### 2.8. Measurement of Bladder ICCs Intracellular Free Ca^2+^ Concentration, [Ca^2+^]i

Preparations were loaded in DMSO containing 5 *μ*mol/L fura-2 AM (Dojindo Laboratories, Japan) for 40 min at 37°C. They were washed three times in PBS and de-esterified for 40 minutes at 37°C before the experiment. Varioskan flash (Thermo Fisher Scientific) was used for measuring [Ca^2+^]i concentration. Intensity ratios at 340 and 380 nm excitation (*F*_340_/*F*_380_) were calibrated using an in vitro method and converted into Ca^2+^ concentration according to the equation, [Ca^2+^]i = *K*_*d*_ ((*R* − *R*_min⁡_)/(*R*_max⁡_ − *R*)), where *K*_*d*_ represents the dissociation constant with a value of 224 nM, *R* represents *F*_340_/*F*_380_, and *R*_min⁡_ and *R*_max⁡_ represent intensity ratios in 0 Ca^2+^ (added 0.1 M EGTA) and saturating Ca^2+^ (0.1% Triton X-100 and 0.1 M CaCl_2_). 

### 2.9. Data Analysis

All data were expressed as mean ± SD. Statistical significance was tested using one-way analysis of variance (ANOVA) and post hoc tests, with values of *P* < 0.05 considered significant.

## 3. Results

### 3.1. Effects of Acupuncture Treatment on Urodynamics

#### 3.1.1. Effects of Acupuncture Treatment on the Detrusor Unstable Contraction Frequency in Bladder Filling Period

No significant differences in body weight were observed among four groups before and after treatment. (*P* = 0.227 before treatment, *P* = 0.77 after treatment). 

**Figure 1:**
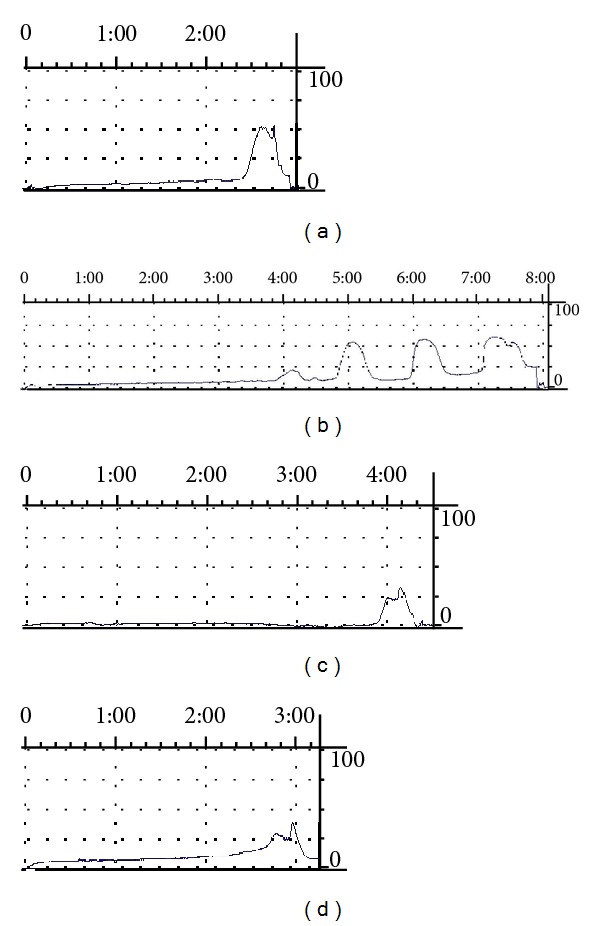
The original tracing of cystometrogram after treatment. (a) Normal control group (b) model group, and (c) acupuncture group; (d): Glivec group.

**Figure 2:**
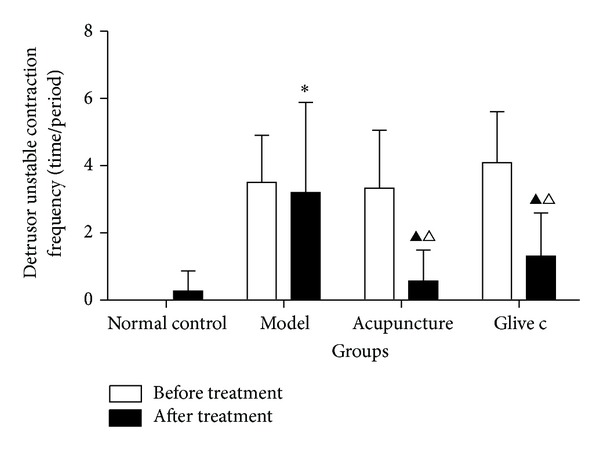
The detrusor unstable contraction frequency in bladder filling period before and after treatment for normal control, model, acupuncture, and Glivec groups. ▲ after treatment versus before treatment *P* < 0.05. ∗ versus normal control group *P* < 0.05. △ versus model group *P* < 0.05.

In cystometrograms performed six weeks after the operation, compared with normal control group (0.00 ± 0.00, *n* = 13), the detrusor unstable contraction frequency (time/period) in bladder filling period was significantly increased in the model group (3.50 ± 1.40, *n* = 15) (*P* = 0.00, *P* > 0.05), acupuncture group (3.33 ± 1.73, *n* = 15) (*P* = 0.00, *P* > 0.05), and Glivec group (4.09 ± 1.52, *n* = 16) (*P* = 0.00, *P* > 0.05), but no significant difference among model group, acupuncture group, and Glivec group (*P* = 0.74, *P* = 0.24, *P* = 0.13, *P* > 0.05). Comparison before and after treatment, there was no difference in the normal control group (*P* = 0.13, *P* > 0.05) and model group respectively, (*P* = 0.64, *P* > 0.05), and decreased in the acupuncture group (*P* = 0.00, *P* < 0.05), and Glivec group respectively, (*P* = 0.00, *P* < 0.05). Comparison among groups after treatment (*F* = 9.805, *P* = 0.00, *P* < 0.05), normal control group (0.27 ± 0.60) acupuncture group (0.57 ± 0.92), Glivec group (1.31 ± 1.29) were less than model group (3.20 ± 2.68) (*P* = 0.00, *P* = 0.00, and *P* = 0.00, *P* < 0.05), and there were no significant difference among the normal group, acupuncture group and Glivec group (*P* = 0.63, *P* = 0.09, and *P* = 0.20, *P* > 0.05) (Figures 1 and 2). 

#### 3.1.2. Effects of Acupuncture Treatment on the Detrusor Unstable Contraction Maximum Pressure in Bladder Filling Period

**Figure 3:**
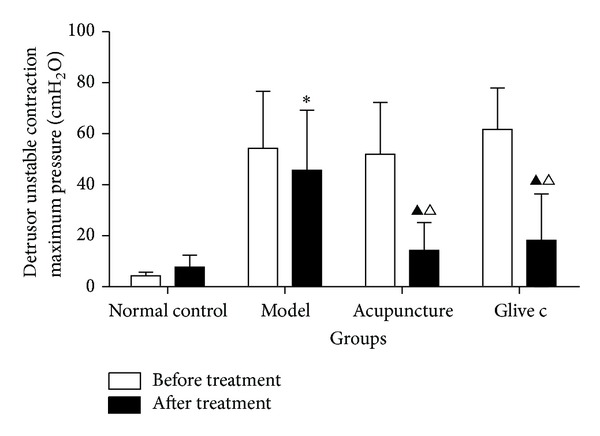
The detrusor unstable contraction maximum pressure in bladder filling period before and after treatment for normal control, model, acupuncture, and Glivec groups. ▲ after treatment versus before treatment *P* < 0.05. ∗ versus normal control group *P* < 0.05. △ versus model group *P* < 0.05.

In cystometrograms performed six weeks after the operation, compared with the normal control group (4.32 ± 1.35, *n* = 13), the detrusor unstable contraction maximum pressure (cmH_2_O) in bladder filling period was significantly raised in the model group (54.27 ± 22.39, *n* = 15) (*P* = 0.00, *P* > 0.05), acupuncture group (51.95 ± 20.33, *n* = 15) (*P* = 0.00, *P* > 0.05), and Glivec group (61.69 ± 16.28, *n* = 16) (*P* = 0.00, *P* > 0.05), but no significant difference among model group, acupuncture group, and Glivec group (*P* = 0.72, *P* = 0.24, and *P* = 0.13, *P* > 0.05). in a Comparison before and after treatment, there was no difference in the normal control group (*P* = 0.06, *P* > 0.05) and model group respectively (*P* = 0.348, *P* > 0.05), and decreased in the acupuncture group (*P* = 0.00, *P* < 0.05) and Glivec group respectively (*P* = 0.00, *P* < 0.05). Comparison among groups after treatment (*F* = 15.305, *P* = 0.00, *P* < 0.05), normal control group (7.68 ± 4.69) and acupuncture group (14.29 ± 10.94), Glivec group (18.24 ± 18.10) were lower than model group (45.69 ± 23.55) (*P* = 0.00, *P* = 0.00, and *P* = 0.00, *P* < 0.05), and there were no significant difference among normal group, acupuncture group, and Glivec group (*P* = 0.29, *P* = 0.09, and *P* = 0.50, *P* > 0.05) (Figures 1 and 3). 

### 3.2. Effects of Acupuncture Treatment on Bladder HCN2 mRNA and Protein

#### 3.2.1. Expression of HCN2 mRNA in Bladder Detected With RT-PCR

**Figure 4:**
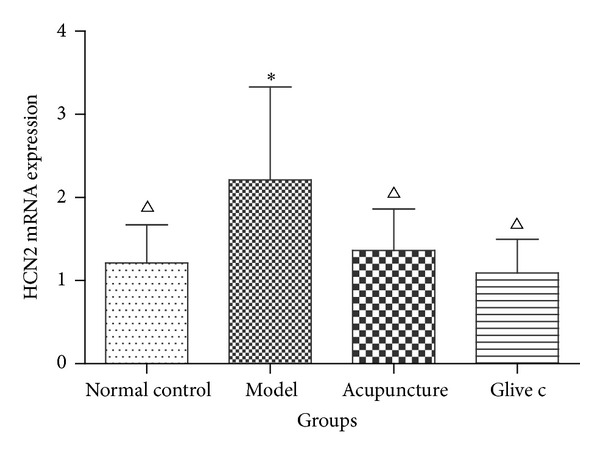
Relative expression level of the HCN2 mRNA in bladder determined by RT-PCR. ∗ versus normal control group *P* < 0.05. △ versus model group *P* < 0.05.

The expression of HCN2 mRNA in bladder was significantly different among groups (*F* = 3.788, *P* = 0.024, *P* < 0.05). Model group (2.21 ± 1.12, *n* = 7) is significantly higher than normal control group (1.21 ± 0.46, *n* = 7) (*P* = 0.01, *P* < 0.05), acupuncture group (1.36 ± 0.50, *n* = 6) (*P* = 0.04, *P* < 0.05), and Glivec Group (1.09 ± 0.41, *n* = 7), (*P* = 0.00, *P* < 0.05) (Figure 4).

#### 3.2.2. Expression of HCN2 Protein in Bladder Detected by Western Blotting

**Figure 5:**
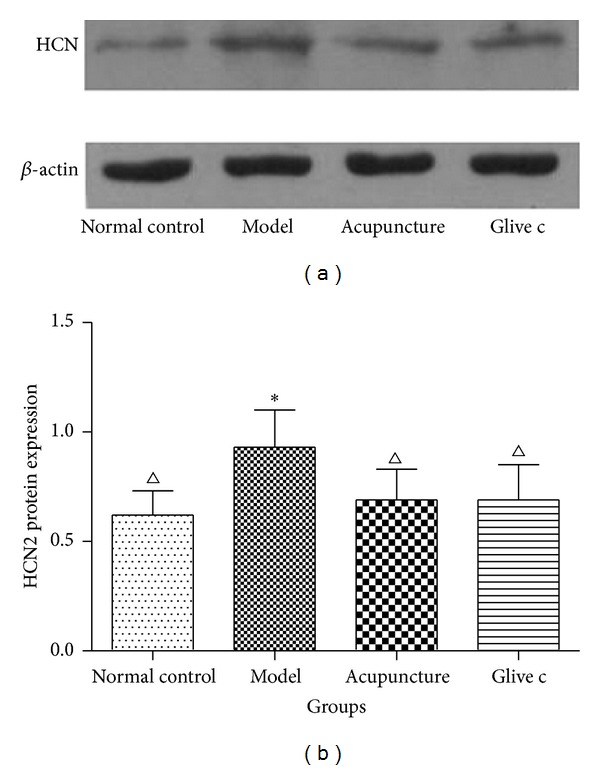
Relative expression level of the HCN2 protein in bladder determined by Western blotting. ∗ versus normal control group *P* < 0.05. △ versus model group *P* < 0.05.

Similar to the result from RT-PCR, the expression of  HCN2 protein in bladder was different among groups (*F* = 3.394, *P* = 0.05). Model group (0.93 ± 0.17, *n* = 4) was increased compared with normal control group (0.62 ± 0.11, *n* = 4) (*P* = 0.01, *P* < 0.05), and significantly decreased in the acupuncture group (0.69 ± 0.14, *n* = 4) (*P* = 0.04, *P* < 0.05) and Glivec group (0.69 ± 0.16, *n* = 4) (*P* = 0.04, *P* < 0.05) (Figure 5). 

### 3.3. Effects of Acupuncture Treatment on Bladder ICCs Distribution and Quantity

**Figure 6:**
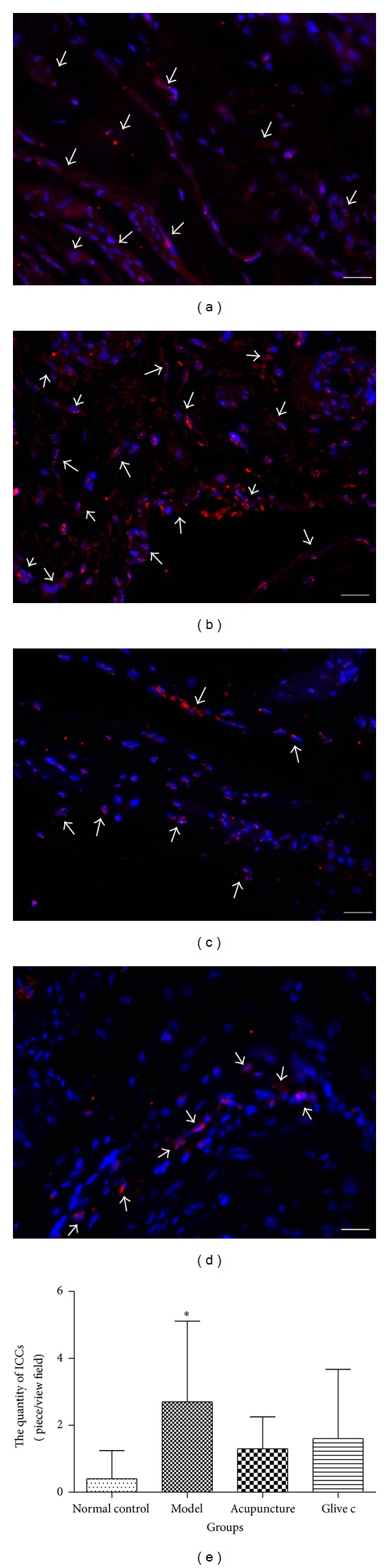
Changes of distribution and quantity in bladder ICCs. ((a), (b), (c), and (d)) Immunofluorescence of c-Kit in bladder. Nucleus stained by DAPI (blue), c-Kit (red) in urothelium, suburothelia, and muscle layer. c-Kit-positive ICCs (↑). (a) Normal control group, (b) model group, (c) acupuncture group, and (d) Glivec group. Scale bar is 50 *μ*m. (e) Comparisons of relative quantity of ICCs in bladder after treatment. ∗ versus normal control group *P* < 0.05.

To detect the ICCs in rat bladder, fluorescent staining method was applied, c-Kit-positive ICCs (red) were found in rat urothelium, suburothelium, and muscle layer by fluorescence microscope. Bladder ICCs were found in detrusor muscle layers that were mainly located along the boundary of smooth muscle bundles and between muscle bundles. The quantity of ICCs was counted in every visual field and 10 visual fields were observed by random in every group. The result showed a significant difference among groups (*F* = 3.086, *P* = 0.039, *P* < 0.05). Model group (2.70 ± 2.41) was more than normal control group (0.40 ± 0.84) (*P* = 0.01, *P* < 0.05), but there were no difference compared with acupuncture group (1.30 ± 0.95) (*P* = 0.08, *P* > 0.05) and Glivec group (1.60 ± 2.07) (*P* = 0.16, *P* > 0.05) (Figure 6).

### 3.4. Effects of Acupuncture Treatment on the Quantity of HCN2 Channel in Bladder ICCs

**Figure 7:**
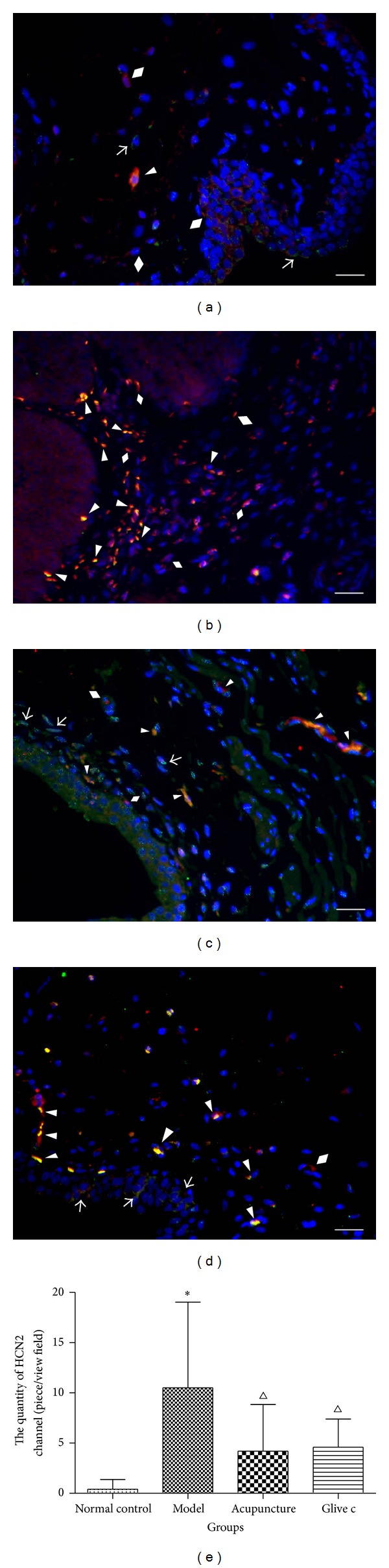
The quantity of HCN2 channel in bladder ICCs. ((a), (b), (c), and (d)) Double-labeled immunofluorescence of HCN2 and c-Kit in bladder. Nucleus stained by DAPI (blue), c-Kit (green), and HCN2 (red) staining in urothelium, suburothelium, and muscle layer. Some ICCs were c-kit positive (↑), some were HCN2 positive (◆), and some were double labeled (▲). (a) Normal control group, (b) model group, (c) acupuncture group, (d) Glivec group. Scale bar is 50 *μ*m. (e) Comparisons of relative quantity of HCN2 channel in bladder ICCs after treatment. ∗ versus normal control group *P* < 0.05. △ versus model group *P* < 0.05.

The relationship between bladder ICCs and HCN2 channel was investigated by double immunofluorescence experiments using c-Kit antibody followed by the antibody protein HCN2, known to be the important features of the pace maker. An relationship was noticed that the HCN2 staining was observed in c-Kit positive ICCs within the individual cell bodies, the HCN2 and c-Kit immunostaining were uniformly present throughout the cytoplasm. HCN2 immunostaining ICCs were also counted in every visual field and 10 visual fields were observed by random in every group. The results revealed a significant difference among these groups (*F* = 6.756, *P* = 0.00, *P* < 0.05), the quantity of HCN2 positive ICCs in model group (10.50 ± 8.53) was significantly increased compared with normal control group (0.40 ± 0.97) (*P* = 0.00, *P* < 0.05), and was less distributed in acupuncture treatment (4.20 ± 4.64) (*P* = 0.01, *P* < 0.05) and Glivec group (4.60 ± 2.80) (*P* = 0.01, *P* < 0.05) (Figure 7). 

### 3.5. Effects of Acupuncture Treatment on Bladder ICCs Intracellular Free Ca^2+^ Concentration

**Figure 8:**
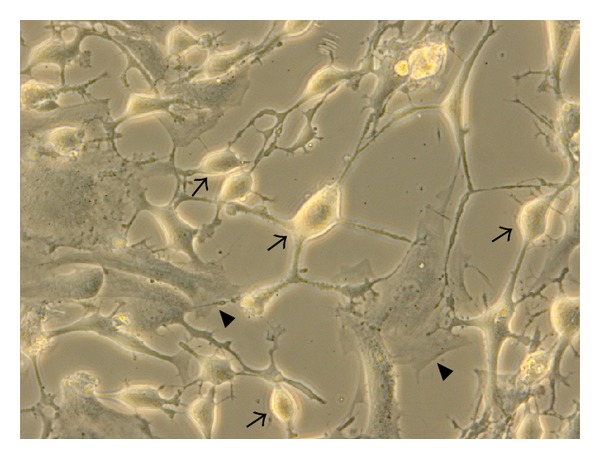
The morphology of ICCs (↑) and detrusor cell (▲) under inverted microscope (×400).

**Figure 9:**
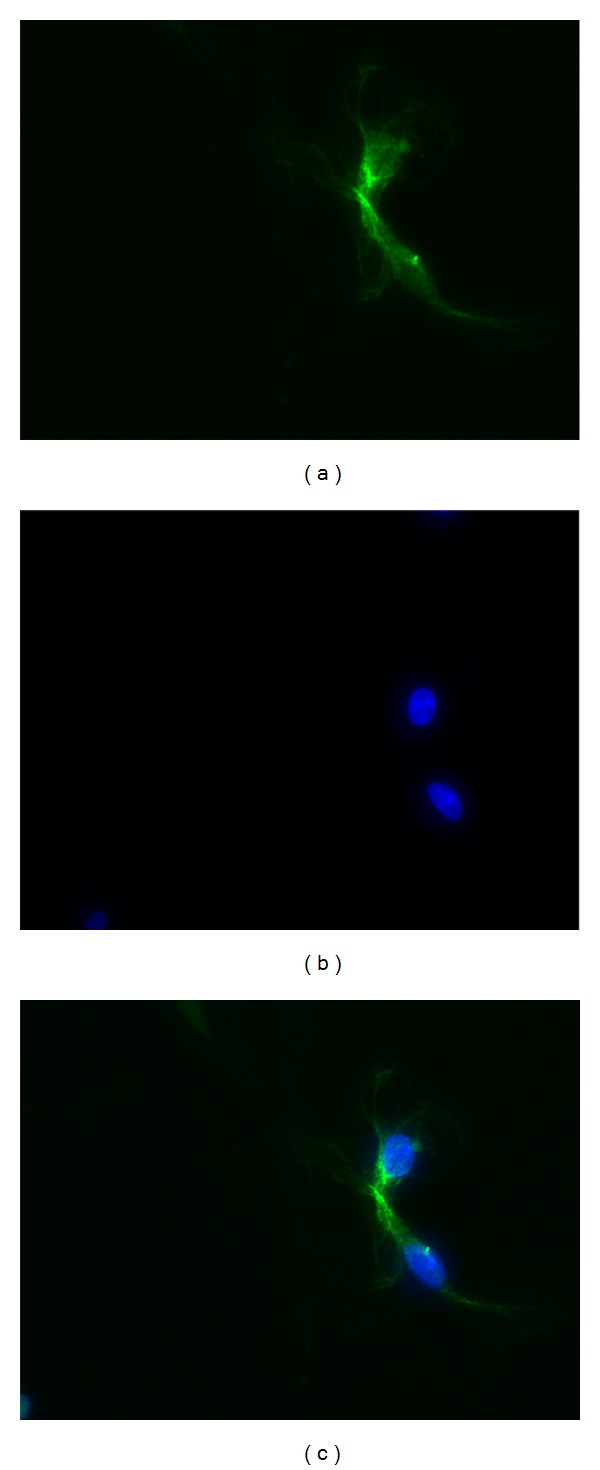
The c-Kit immunofluorescence of ICCs under Inverted Fluorescence Microscope (×400). (a) c-Kit-positive ICCs (green). (b) Nucleus stained by DAPI (blue). (c) Merge.

Cultured cells were observed under inverted microscope. The morphology of ICCs was spindle-shaped, with several branches emanating from a central soma, and connected with neighboring cells, showing networks (Figure 8). The ICCs in the cultures were morphologically distinct from the smooth muscle cells and their identity was confirmed with immunocytochemistry using primary antibodies against c-Kit (Figures 9(a), 9(b), and 9(c)).

**Figure 10:**
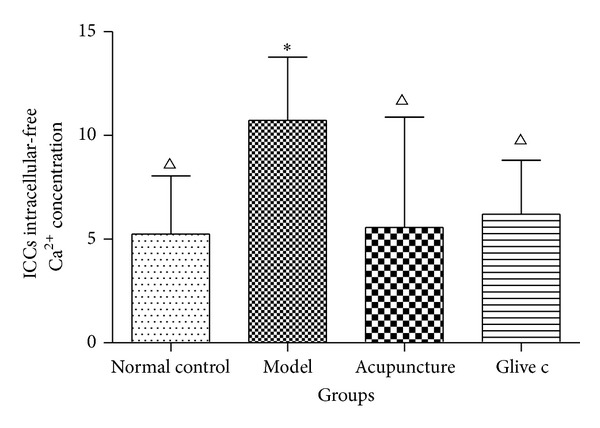
Comparisons of relative bladder ICCs intracellular-free Ca^2+^ concentration among four groups after treatment. ∗ versus normal control group *P* < 0.005, △ versus model group *P* < 0.005.

The ability of rat bladder ICCs to respond to the acupuncture stimulation and Glivec was investigated in primary cultured and fresh cells, loaded with Ca^2+^ indicator fura-2 AM. Varioskan flash (ThermoFisher Scientific) was used for measuring [Ca^2+^]i concentration, every well in 96-well plates was determined on 5 regions of interest, and 40 regions were selected in each group. According to the equation, [Ca^2+^]i = *K*_*d*_ ((*R* − *R*_min⁡_)/(*R*_max⁡_ − *R*)), to convert into Ca^2+^ concentration. The intracellular free Ca^2+^ concentration in bladder ICCs of model group (10.73 ± 3.05) was higher as compared to normal control group (5.24 ± 2.80), (*P* = 0.00, *P* < 0.05); acupuncture stimulation (5.57 ± 5.31) and Glivec bladder irrigation (6.21 ± 2.60) was significantly lower the OAB rats bladder ICCs intracellular Ca^2+^ concentration (*P* = 0.00, *P* = 0.00, *P* < 0.05) (Figure 10).

## 4. Discussion

This study demonstrated that acupuncture stimulation suppressed bladder overactivity, down-regulated the expression of HCN2 mRNA and protein in bladder, reduced the quantity of HCN2 channel in bladder ICCs, and also decreased bladder ICCs intracellular free Ca^2+^ concentration. This is the first evidence that acupuncture stimulation regulates the excitability of  bladder ICCs in treatment of overactive bladder myogenic mechanism.

The points of stimulation in this study are the classical points BL32 and BL35 corresponding to sacral vertebrae S_2_ level and coccyx, which belongs to the Bladder Meridian of Foot Taiyang in Traditional Chinese Medicine [21]. The clinical studies show that acupuncture stimulation to the sacral vertebrae increases bladder capacity and suppresses overactive bladder [22, 23]. In our previous clinical studies, we found that electroacupuncture with continuous wave, 30 Hz, 1 mA, 20 min, 3 days stimulation could suppress bladder unstable contraction frequency, and decrease detrusor maximum pressure in the bladder filling period thereby suppressing bladder overactivity. Therefore, we used the same therapy through the experiment. 

ICCs have been widely recognized as playing key roles in the initiating contractile activity, mediating neurotransmission, conducting electrical impulses, and acting as a stretch sensor [24]. The spontaneous action potentials identified in the ICCs indicate that changes in the excitability of this cell lead to a series of contractile abnormality. The number of ICCs was increased in overactive bladder, the changes in ICCs may account for pathologically increased signal transmission between cells [25]. The clinical significance of bladder ICC in bladder dysfunction has been investigated by studies of tissue from patients with unstable bladders which reportedly have larger numbers and density of distribution of ICC than control samples, Glivec (imatinib mesylate) 10^−5 ^M, improved bladder capacity, compliance, voided volumes, urinary frequency, and reduced contraction thresholds and spontaneous activity [26]. Our results show that the quality of c-Kit-positive bladder ICCs in OAB rats was significantly increased compared with normal control rats, which was consistent with the results of above studies. However, the quatity was not significantly reduced by acupuncture stimulation and Glievc 10^−5 ^mol/L bladder irrigation. 

HCN channels are active at resting membrane potential and thus are believed to participate in autorhythmicity and excitability. Immunofluorescence and Western blotting revealed that HCN1, HCN2, HCN3, and HCN4 channels were all present in the ICCs of the human bladder [27]. HCN1, 2, 4 subtypes play important role in modulating bladder excitability by controlling the kit-positive ICC-like cells. HCN channel, and its Ih current may take part in the modulation of cell excitation and might control the excitability of bladder. HCN channel, as a potential peacemaker, was involved in the regulation of bladder excitation, presumably via bladder ICC-like cells [28]. In our study, immunohistochemistry was also used to detect HCN2 channel. The quality of HCN2 in ICCs in OAB rats was significantly increased compared with normal control rats, and significantly reduced by acupuncture stimulation and Glievc 10^−5^ M bladder irrigation. The results from RT-PCR and western blotting analysis revealed that HCN2 subtype was detected in bladder, and its mRNA and protein expression were increased in OAB rats, suggesting that there are some changes in the HCN2 channel in the pathologic conditions, with the excitability enhancement of HCN2 channel in bladder. HCN2 involved in the suppressing effect of acupuncture stimulation on bladder unstable contraction, acupuncture stimulation and Glivec 10^−5 ^M bladder irrigation down-regulated the HCN2 mRNA and protein expression, suggesting that HCN channel in ICCs may be the new therapeutic target for OAB. 

Modulation of cytoplasmic Ca^2+^ concentration is a mechanism common to signal transduction pathways regulating many cellular phenomena, the role of intracellular Ca^2+^ is of particular importance, as it is the main determinant of detrusor contractile activity. Spontaneous, autonomous cellular activity-Ca^2+^ and membrane potential oscillations, originate from detrusor smooth muscle in human bladders, mediated by extracellular Ca^2+^ influx and intracellular release [29]. Meanwhile, these pace maker cells ICCs generate the repetitive Ca^2+^ transients that activate inward currents that spread through the gap junctions to provide the depolarizing signal that triggers contraction through opening L-type voltage-operated channels (VOCs) allowing external Ca^2+^ to flood into the cell to trigger contraction [30]. In this study, we revealed that basal intracellular free Ca^2+^ concentration was elevated in Bladder ICCs from overactive bladders and was significantly decreased through acupuncture stimulation and Glivec, which suggest that acupuncture might have affected the membrane potential and made it difficult to induce Ca^2+^ influx, and the L-type calcium channel opened to trigger the action potential.

## 5. Conclusions

A systematic profile of the effects of acupuncture stimulation on bladder overactivity and the excitability of bladder ICCs were shown in the present study. The results show that acupuncture stimulation suppressed bladder overactivity, down-regulated the expression of HCN2 mRNA and protein in bladder, and reduced the quality of HCN2 chanel in bladder ICCs. Furthermore, acupuncture stimulation also decreased bladder ICCs intracellular-free Ca^2+^ concentration. The result may help to provide a scientific foundation to build the clinical acupuncture treatment in OAB.
